# The Conners Continuous Performance Test CPT3^™^: Is it a reliable marker to predict neurocognitive dysfunction in Myalgic encephalomyelitis/chronic fatigue syndrome?

**DOI:** 10.3389/fpsyg.2023.1127193

**Published:** 2023-02-27

**Authors:** Judith Fernández-Quirós, Marcos Lacasa-Cazcarra, Jose Alegre-Martín, Ramón Sanmartín-Sentañes, Miriam Almirall, Patricia Launois-Obregón, Jesús Castro-Marrero, Amanda Rodríguez-Urrutia, Jose A. Navarro-Sanchis, J. Antoni Ramos-Quiroga

**Affiliations:** ^1^Myalgic Encephalomyelitis/Chronic Fatigue Syndrome Unit, Division of Rheumatology, Vall d’Hebron Hospital Research Institute Universitat Autònoma de Barcelona, Barcelona, Spain; ^2^Department of Mental Health, Hospital Universitari Vall d’Hebron, Barcelona, Spain; ^3^e-Health Center, Universitat Oberta de Catalunya, Barcelona, Spain; ^4^Group of Psychiatry, Mental Health and Addictions, Vall d’Hebron Research Institute (VHIR), Barcelona, Spain; ^5^Biomedical Network Research Centre on Mental Health (CIBERSAM), Barcelona, Spain; ^6^Department of Psychiatry and Forensic Medicine, Universitat Autònoma de Barcelona, Barcelona, Spain

**Keywords:** neuropsychological test, cognitive impairments, continuous performance test, CPT3^™^, neurocognitive dysfunction, Central Sensitization Syndromes, chronic fatigue syndrome

## Abstract

**Introduction:**

The main objective is to delimit the cognitive dysfunction associated with Myalgic Encephalomyelitis/Chronic Fatigue Syndrome (ME/CFS) in adult patients by applying the Continuous Performance Test (CPT3^™^). Additionally, provide empirical evidence on the usefulness of this computerized neuropsychological test to assess ME/CFS.

**Method:**

The final sample (*n* = 225; 158 Patients/67 Healthy controls) were recruited in a Central Sensitization Syndromes (CSS) specialized unit in a tertiary hospital. All participants were administered this neuropsychological test.

**Results:**

There were significant differences between ME/CFS and healthy controls in all the main measures of CPT3^™^. Mainly, patients had a worse indicator of inattentiveness, sustained attention, vigilance, impulsivity, slow reaction time, and more atypical T-scores, which is associated with a likelihood of having a disorder characterized by attention deficits, such as Attention Deficit Hyperactivity Disorder (ADHD). In addition, relevant correlations were obtained between the CPT3^™^ variables in the patient’s group. The most discriminative indicators of ME/CFS patients were Variability and Hit Reaction Time, both measures of response speed.

**Conclusion:**

The CPT3^™^ is a helpful tool to discriminate neurocognitive impairments from attention and response speed in ME/CFS patients, and it could be used as a marker of ME/CFS severity for diagnosing or monitoring this disease.

## Introduction

Myalgic Encephalomyelitis/Chronic Fatigue Syndrome (ME/CFS) is a complex multisystem disease that presents a chronic course with periods of symptomatic exacerbation frequently related to acute stress. This condition predominantly affects women and is a severe functional disorder (Afari and Buchwald, 2003; Prins et al., 2006). The prevalence is estimated to be between 0.2 and 2.6% of the general population (Reyes et al., 2003; Nacul et al., 2011). The nuclear symptoms are chronic central fatigue (>6 months) and post-exertional malaise with a recovery time longer than 24 h of idiopathic origin. The international diagnostic criteria were established by the Centres for Disease Control (CDC) in Atlanta (Georgia) in 1994 (Fukuda et al., 1994). Furthermore, the heterogeneity of symptoms inME/CFS is studied in clusters from 2003 as a complement to the CDC’s criteria (neurological, muscle, cognitive, neurovegetative, and immunological; Fukuda et al., 1994; Carruthers et al., 2003). In 2011, these criteria were updated, and post-exertional exhaustion was proposed as a disease hallmark (Carruthers et al., 2011). ME/CFS is associated with different comorbid phenomena (anxiety-depressive disorders, fibromyalgia (FM), sicca syndrome, regional myofascial pain syndrome, plantar fasciitis, degenerative or mechanical disk disease, and tendinopathy of the shoulder; Ruiz et al., 2011 Castro-Marrero et al., 2016) that are more prevalent in ME/CFS patients than in non-CFS individuals (Rivera et al., 2006). At present, the most widely accepted hypothesis for the pathogenesis of ME/CFS characterizes it as a genetic-based process with different triggering factors and subsequent neuroimmunology and immunoinflammatory dysfunction (Bassi et al., 2008).

Fatigue is the most common symptom associated with chronic diseases and is experienced as really distressing. Nature fatigue is a subjective state with both physical and psychological elements, and there is a lack of effective treatments for it. New methods are being developed to quantify fatigue and are increasing in clinical settings (Swain, 2000). Fatigue may occur due to a physical or psychological event, or fatigue may cause a physical event. The concept of fatigue appears to be defined by overlapping terms of cognitive or mental fatigue; however, the description for each slightly varies. For example, cognitive fatigue is said to occur when cognitive performance decreases by engaging in tasks requiring sustained activity (Morrow et al., 2015). Mental fatigue has been defined as a subjective feeling of tiredness and inertia that occurs during extended periods of demanding cognitive activity (Badin et al., 2016). Fatigue has also been defined as physiological fatigue, which is described as muscle weakness that may occur due to exercise (Prinsen et al., 2015). There is insufficient evidence examining the relationship between fatigue and cognitive impairments in patients with chronic illnesses. More studies are needed to examine the potential relationships between these two symptoms to develop effective treatments for individuals living with NCDs (Chronic Non-Communicable Diseases; Menzies et al., 2021).

Cognitive deficits are common in ME/CFS patients and limit their quality of life and psychological well-being. The discrepancy between self-reported common cognitive deficits and results obtained by objective neuropsychological tests has been reported (Rasouli et al., 2019). This disparity could be explained by several reasons, mainly methodological (Cockshell and Mathias, 2010), suggesting establishing an appropriate method to avoid it. Patients with higher levels of fatigue, pain, and depression reported greater subjective cognitive difficulties, and those with greater pain were associated with lower objective working memory function. ME/CFS patients primarily had psychomotor speed and attention problems, measured by objective neuropsychological tests (Rasouli et al., 2019). Other studies confirm that cognitive deficits in ME/CFS depend mainly on compromised attention, memory, and reaction time, but motor functioning, vocabulary, reasoning, and global deficits are absent (Cockshell and Mathias, 2010).

Cognitive dysfunction has been described in patients with depression and anxiety, and it is difficult to determine whether this is due to psychopathological comorbidity or fatigue. Slowing information processing speed occurs, especially in complex tasks requiring sustained attention. However, the underlying mechanisms of the manifested cognitive dysfunction remain unclear (Cvejic et al., 2016), advising a need for appropriate neuropsychological assessment tools in this complex disease capable of overcoming such limitations. One study showed that the dysfunction of information processing speed is independent of depressive symptoms in ME/CFS, suggesting that attentional deficits may be primary to memory problems, which could show an underlying neurological basis for the attentional dysfunction in these patients (Santamarina-Perez et al., 2014). Unfortunately, there are currently no commercially available diagnostic tests, specific lab biomarkers, or targeted FDA-approved ME/CFS drugs (Castro-Marrero et al., 2017). For all these arguments, and given the disparity of the published studies, there is no consensus on the results regarding cognitive dysfunction in ME/CFS and its repercussions. Therefore, more research is needed on this symptom in ME/CFS and the detection of the underlying mechanisms. The limitations found in previous studies on this topic must be considered to improve their research and obtain more specific neuropsychological assessment tools to evaluate it (Beaumont et al., 2012). General dysfunction in attention and information processing speed have been suggested as reasons for ME/CFS memory complaints (Deluca et al., 2004; Dickson et al., 2009).

Considering the evidence of neuropsychological dysfunction in ME/CFS patients, this work is carried out using a computerized test that allows comprehensive measurement of different cognitive domains of interest in this disease. Therefore, this is the first study in ME/CFS patients using the CPT3™. This neuropsychological test has been widely used to objectively and comprehensively assess cognitive functioning in patients diagnosed with Attention-Deficit Hyperactivity Disorder (ADHD; Fasmer et al., 2016; Berger et al., 2017).

CPT3™ allows evaluating the participant’s performance in just 14 min, contemplating the “fatigue” variable (the core symptom of this disease) and other multiple cognitive measures. It is not only interesting because of the novelty of introducing this software tool in patients with ME/CFS, but also because it allows the evaluation of multiple indicators of cognitive functioning such as attentional capacity and some executive functions (inhibition and information processing speed) trying to find a differential clinical profile between patients and healthy controls (Kao and Thomas, 2010).

## Method

### Sample

The study subjects were composed of two groups, healthy controls and patients diagnosed with ME/CFS. Patients with a diagnosis of ME/CFS have been recruited consecutively from the Central Sensitization Syndromes (SCC) specialized unit at the Vall d’Hebron University Hospital in Barcelona from July 2021 to March 2022. Inclusion criteria were fulfilled the Fukuda et al. (1994) and Carruthers criteria for ME/CFS(2), age between 18 and 65 years, the subjective clinical manifestation of cognitive impairment according to Carruthers et al. (2003, 2011), understanding and acceptance of participation in the study and signed written informed consent. The research protocol was approved by the ethics committee with this registration code(PR(AG)257/2021). The patients underwent an extensive medical examination by specialist physicians belonging to the Central Sensitization Syndromes (CSS) experienced Unit of the Rheumatologist Department at this hospital. The exclusion criteria were difficulty in understanding and/or completing the self-reported questionnaires, the presence of severe unstable psychiatric disorders (such as psychotic episodes, major depressive episodes, manic episodes, and anorexia nervosa), history of neurological disorders with cognitive impairment (such as severe head-brain trauma), presence of another systemic disease with cognitive alterations and presence of the disease entity Mild Cognitive Impairment (MCI). All patients diagnosed with ME/CFS included in the study were recorded on a data collection sheet in encrypted form and subsequently included in a database. The results obtained from the sample through the CPT3™ software were recorded in an excel table. The minimum sample calculation to demonstrate a statistically significant difference between the mean of the measurements of the cognitive domain variable, information processing speed (HRT), according to Student’s *t*-test, is 92 patients with a confidence level equal to 0.95. Pearson’s coefficient, which measures the variance to the mean, is 20%.

Of the total initial sample, four patients were excluded because of an associated severe psychiatric or neurological comorbidity at that precise moment that could account for some of the ME/CFS symptoms (three had a major depressive episode, and one patient with severe head-brain trauma). In addition, six patients were also excluded because they withdrew on their own initiative, indicating fear of fatigue from the mental overexertion of the test.

### Neuropsychological evaluation

In the first session, the participants were individually examined by the ME/CFS specialist physician and were given questionnaires to fill in. The perception of disabling fatigue, sleep problems, and health-related quality of life using self-administered questionnaires: the fatigue impact scale FIS40 (Fisk et al., 1994), FIS8 (Fisk and Doble, 2002), PSQI (Verster et al., 2008), the Short Form Health Survey (SF-36; Alonso et al., 1995), the Symptom Checklist-90-revised (SCL 90 R) psychological inventory (McGregor et al., 1997) and hospital anxiety and depression scale (HAD; Castresana et al., 1995). The interviews were conducted by two internists and one rheumatologist physician skilled in diagnosing and treating this syndrome.

The neuropsychological evaluation was performed in a second appointment. It consisted of a single standardized individualized examination in which cognitive functioning was assessed using the latest version of the 3rd Conners Continuous Performance Test CPT3™ (Kao and Thomas, 2010). The participants had never been administered the CPT3™ to avoid familiarity. They were required to respond when any letter except “X” appeared on the monitor. CPT3™ was performed from 9:00 am to 2:00 pm, always in the same medical office under standard temperature, noise, and lighting conditions, and with mobile turned off to avoid interference. It was decided to establish this time slot for the evaluation due to several reasons (for example, attentional, and cognitive performance is not as optimal at certain times of the day, and fatigue accumulates as the day progresses both in the clinical population and in healthy controls as a general rule). The evaluation protocol was not modified for any patient.

CPT3™ is a computerized task of continuous execution based on performance to assess primarily attention-related impairments in individuals aged 8 years and older. The administration time is 14 min, during which the subject must maintain attention to perform this task. There are six blocks of trials, with three sub-blocks, each consisting of 20 trials. Each stimulus appears on the screen with varying time frequencies (inter-stimulus intervals from 1, 2, and 4 s), which allows for comparison of the subject’s attention and response speed according to the time intervals. This software makes it possible to compare the changes in performance that may be experienced in separate segments (block measurement), thus checking whether the level of vigilance fluctuates during this simple task. Its duration allows for controlling the “fatigue effect” on cognitive performance, a key aspect to consider in patients diagnosed with CFS. Before starting, some instructions must be given. Individuals are seated in front of a computer. Each participant is required to respond (pressing the space bar on the keyboard) when any letter (target stimuli), except the letter “X” (non-target stimuli), appears on the screen. It is essential to warn them that they must keep responding until the end of the test to obtain the computer-generated reports describing the respondent’s performance in detail. Once the subjects have understood the instructions, they perform the test. Each participant’s final report was recorded in an Excel spreadsheet. Subsequently, the neurocognitive profile of the whole sample was studied by analyzing all the results obtained (Kao and Thomas, 2010).

### Main features of the CPT3™

The CPT3™ test offers already standardized and raw scores to determine not only the overall performance of the evaluation but also specify different types of attention deficits (e.g., inattentiveness, impulsivity, sustained attention, vigilance) and response speed, allowing a comprehensive assessment. So, multiple features are measured in The CPT3™.

A brief description of the main measures used in this study is exposed below:

- Response speed: to obtain information performance about motor reaction time and information processing speed measured by the software (HRT, HRTSD, HRT block change, …).
- Impulsivity: is an indicator of the response inhibition capacity of the evaluated and includes a faster than normal HRT and a higher than average rate of commissions and/or perseverations.
- Inattentiveness: scores about focused attention. This indicator relates to poor detectability, a high percentage of omissions and commissions, a slow HRT, and high levels of inconsistency in response speed.
- Sustained attention: is defined as the respondent ability to maintain attention as the administration progresses. A decrease in sustained attention across time is captured by atypical slowing in the respondent’s HRT and by increases in omissions and commissions in later blocks of the administration.
- Vigilance: relates to the respondent’s performance at varying levels of stimulus frequency and is defined by the respondent’s ability to maintain a performance level even when the task rate is slow. It is captured by changes in the respondent’s HRT, as indicated by the variables HRT ISI.
- Detectability: discrimination between non-targets and targets.
- Omissions: are missed targets and are generally an indicator of inattentiveness.
- Commissions: are incorrect responses to non-targets. High commission error rates may indicate either inattentiveness or impulsivity, depending on the respondent’s HRT.
- Perseverations are responses made in less than 100 ms following the presentation of a stimulus. Perseverations may be related to impulsivity or an extremely liberal response style.
- Hit reaction time (HRT): the mean response speed, measured in milliseconds, for all non-preservative responses made during the entire administration. An atypically slow HRT may indicate inattentiveness but may also result from a conservative response style. So, HRT is also affected by response style.
- HRT standard deviation (HRT SD): measures the consistency of response speed for the entire administration. A high HRT SD indicates a greater response speed inconsistency, sometimes indicative of inattentiveness.
- Variability: is a measure of response speed consistency; however, Variability is a “within respondent” measure (i.e., the amount of variability the respondent showed in 18 separate sub-blocks of the administration with the overall HRT SD score). High response speed variability indicates that the respondent’s attention and information processing efficiency varied throughout the administration.
- HRT block change: the slope of change in HRT across the six blocks of the administration. A positive slope indicates decelerating HRT as the administration progressed; a negative slope indicates accelerating HRT; a flat slope indicates no change in HRT.
- HRT Inter-Stimulus Interval (ISI) change: is the slope of change in reaction time across the three ISIs (1, 2, and 4 s). A positive slope indicates decelerating HRT at longer intervals, whereas a negative slope indicates accelerating HRT at longer intervals.

Based on the respondent’s pattern and scores in each attentional dimension, the software identifies the presence and severity of the kinds of attention problems the respondent is most likely having. Therefore, CPT3™ provides in the outcome report the likelihood of having a disorder characterized by attention deficits, such as ADHD (Kao and Thomas, 2010).

In this research, a categorical dichotomous variable (YES/NO; obtained from the assessment report) is analyzed to indicate whether those evaluated have this probability of having an attentional disorder (YES indicates moderate to high possibility).

### Statistical method

#### Categorical features

For each feature, a 2×2 contingency table is set up to perform the chi-squared test of independence for the groups defined as group C (control group of healthy patients) and group P (diagnosed group of ME/CFS patients). The significance threshold has been defined as a *p*-value <0.05. Cramer’s V is an effect size measurement for the chi-square test of independence. It measures how strongly two categorical fields are associated. The degree of freedom (df) is 1, and if Cramer’s value >0.1 is considered small-medium, >0.30 is considered medium-large, and > 0.50 is considered significant (Kim, 2017). Cramer’s V was based on Pearson’s chi-squared statistic and was published by Harald Cramer in 1946 (Doob, 1946).

#### Numerical features

The Shapiro–Wilk test and D′Agostino’s K-squared test are two of the most commonly used hypothesis tests to analyze normality. In both tests, the null hypothesis is that the data comes from a normal distribution. The *p*-value of these tests indicates the probability of obtaining data like the observed data if they came from a population with a normal distribution with the same mean and variance as the observed data. The threshold is a *p*-value <0.05 as sufficient evidence to reject normality. The purpose is to verify the conditions of parametric methods for using the *t*-test. For features with a non-normal distribution, the test of independence used is the Mann–Whitney *U*-test for independent samples. The analysis is continued by calculating the arithmetic means of each variable and the graphical calculation using the box plot analysis.

#### Relationship graph between features

The relationships between the features have been analyzed using graph theory. A graph is a collection of nodes (also called vertices) joined together in pairs by edges (undirected) or arcs (directed; Newman, 2018). The graph structure allows us to capture the pattern of interactions between the nodes (individuals or entities). Graph (or network) analysis is used to study relationships between individuals to discover knowledge about global and local structures. The study of structure networks helps to decide the optimal order (Hagberg et al., 2008). In this work, the graph nodes are defined as all features, and the edges are defined as moderate or strong correlations between nodes (features). The correlation between two features is represented by $\mathrm{corr}\left(i,j\right)$ , and Spearman correlation is defined as moderate or strong if $\mathrm{corr}\left(i,j\right)\geq 0.5$ (Suchowski, n.d.) in case of direct correlation. It has been created an $\mathrm{edge}\left(i,j\right)$ if $\mathrm{abs}\left(\mathrm{corr}\left(i,j\right)\right)\geq 0.5$ in order to include direct and indirect correlation. This analysis used the values of the P-group diagnosed by ME/CFS.

#### Random forest algorithm

Random forest is a widespread algorithm for classification and offers the importance of feature values. All classified samples from the two defined groups are used to obtain a model. This model classifies future samples according to both categorical and numerical CPT3 scores. The metric used was accuracy. Features importance are computed as the mean and standard deviation of accumulation of the impurity decrease within each tree. It informs which feature has greater power to classify (Liaw and Wiener, n.d.).

## Results

The sample size’s value was based on a *t*-student analysis for two populations, ME/CFS patients (P) and healthy controls (C). Distributions of CPT3™ variables were examined before analysis. Sample Analysis is reported in Table 1.

**Table 1:** Sample analysis.

	C (Control group)	P (CFS Diagnosed group)
*n* (225)	67	158
Age mean (std)	44.82 ± 14.26	51.40 ± 8.11
Females	53 (79.1%)	145 (91.77%)
Males	14 (20.9%)	13 (8.23%)

There are 17 features analyzed, six categorical and 11 numerical. For categorical features, the Chi-Squared test of independence is used related to C (Control group) or P (Diagnosed group). Tests of independence (Chi-Square, T-Student, and Mann–Whitney) indicate if there are significant differences between the two populations of the study. The results are shown in Tables 2, 3.

**Table 2:** Results from numerical features from CPT3™ by groups.

Feature	Control group (C)		Diagnosed group (P)		Independent test
	Mean	STD	Mean	STD	*p*-Value
*t*-student test (for normal distribution variables)					
Res.Styl N	47.82	8.05	48.95	11.21	0.39644
Detectab N	46.94	8.67	57.30	11.68	0.00000
Mann–Whitney *U* Test (for not normal distribution variables)					
Omission N	46.54	3.60	55.28	13.85	0.00000*
Commiss N	48.64	8.76	57.11	12.18	0.00000*
Persever N	48.63	5.93	58.81	16.25	0.00045*
HRT N	47.33	12.22	53.36	13.24	0.00011*
HRT SD N	46.54	7.56	61.65	14.70	0.00000*
Variabil N	45.01	6.48	55.61	11.73	0.00000*
HRT Blo C N	49.76	8.30	48.03	13.38	0.457403*
HRT ISI Ch N	50.42	9.04	50.63	14.23	0.949235*

Mean values according to the type of patient in the P group (CFS patient) or the Control group. The independence test was performed using Student’s *t*-test if the values correspond to a normal distribution or Mann–Whitney *U* test otherwise and marked with an asterisk “*.” For values with a *p*-value of less than 0.05, the hypothesis that group membership predetermines the outcome of the variable studied is considered to be accepted.

**Table 3:** Chi-square test of independence results.

Feature	Chi square value	*p*-Value	Cramer’s*
Attention deficit	43.14	0.00000	0.44
Inattentiveness	50.45	0.00000	0.47
Impulsivity	6.27	0.01225	0.17
Sustained attention	3.87	0.04920	0.13
Vigilance	12.33	0.00045	0.23
Gender	6.00	0.01430	0.16

* Attention deficit, inattentiveness, impulsivity, sustained attention, and vigilance was all significant. Gender was also found to be significant. Cramer, greater than 0.30, in Inattentiveness (0.47 Cramer’s *), Attention Deficit (0.43 Cramer’s *), and Vigilance (0.23 Cramer’s *) are significant (medium relation if Cramer >0.30 with df = 1). Significant differences indicate that the samples come from different populations. Consequently, it can be inferred that the significant variables predict the group the participant belongs to.

As a result, a pattern of attention deficit is obtained in the ME/CFS group when recording as a categorical dichotomous variable (YES/No) the probability of having a disorder characterized by attention deficits, such as ADHD (YES, including moderate to high likelihood of having it). With T-Student (if normality), the detectability feature is significant. With Mann–Whitney *U* Test (not normality), all variables are significant except HRT Blo C and HRT ISI Ch N, which are not.

**Figure 1:**
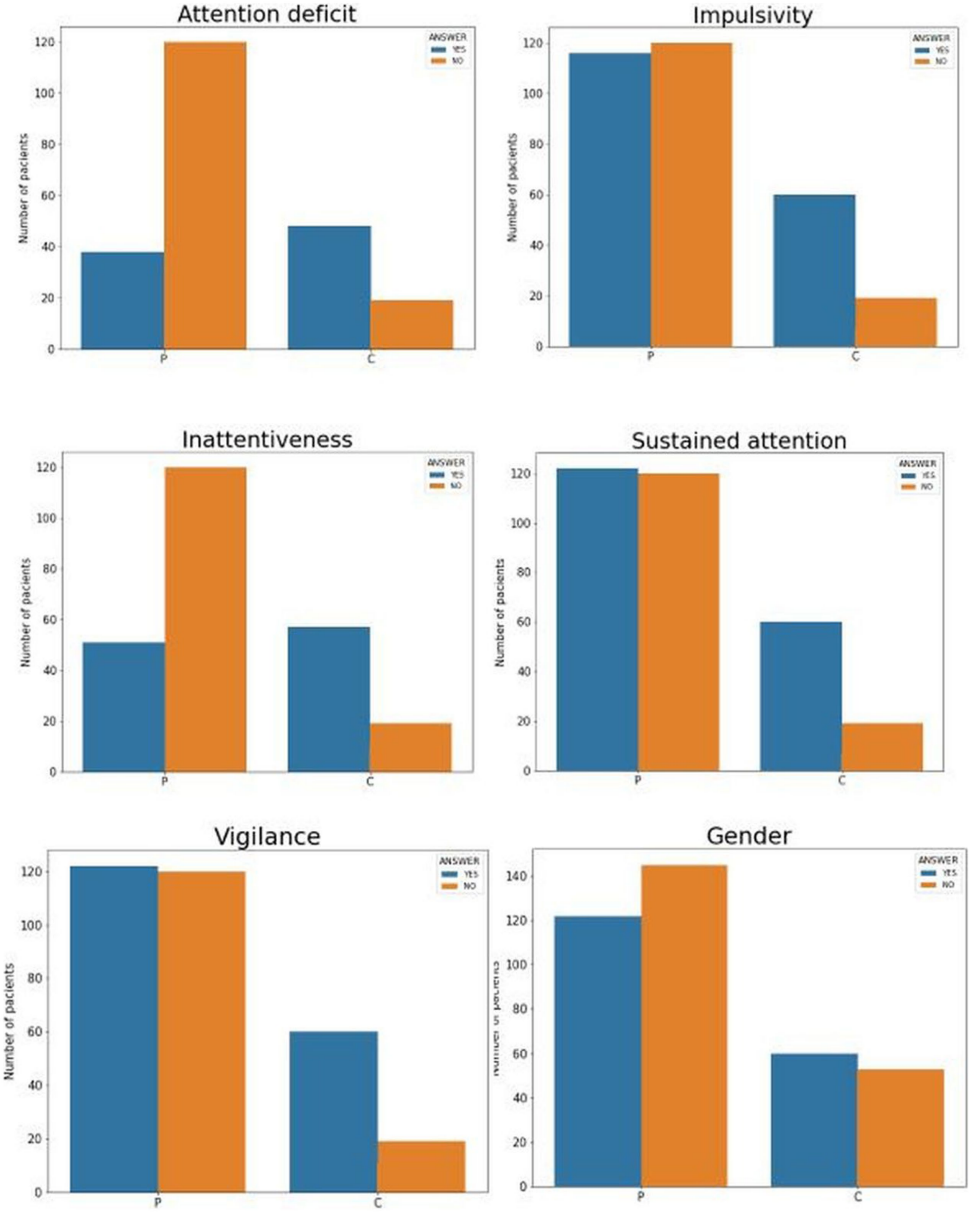
Graph of the contingency tables by groups and normalized, where each bar corresponds to the percentage of each value by groups. Each drawing represents for each dichotomous variable the group differences, with C being the control group and P the diagnosed group. The following acronyms mean the six dichotomous variables: DAT, attention deficit; INAT, inattentiveness; IMPUL, impulsivity; AT.SOST, sustained attention; VIGIL, vigilance; and finally, Gender.

The categorical variables are dichotomous, with values {1,2}, the plots showing whether significant differences exist due to belonging to one group or another. The graph in Figure 1 shows the percentage of the value of each variable according to the group. For example, it can be seen that in the first graph, the D.AT variable (attention deficit) is positive (value 2) in 30% of the control group and over 70% in the group of diagnosed patients. In this and the inattentiveness (INAT) variable, the differences are marked and reflected in the subsequent Chi-Square test of independence.

**Figure 2:**
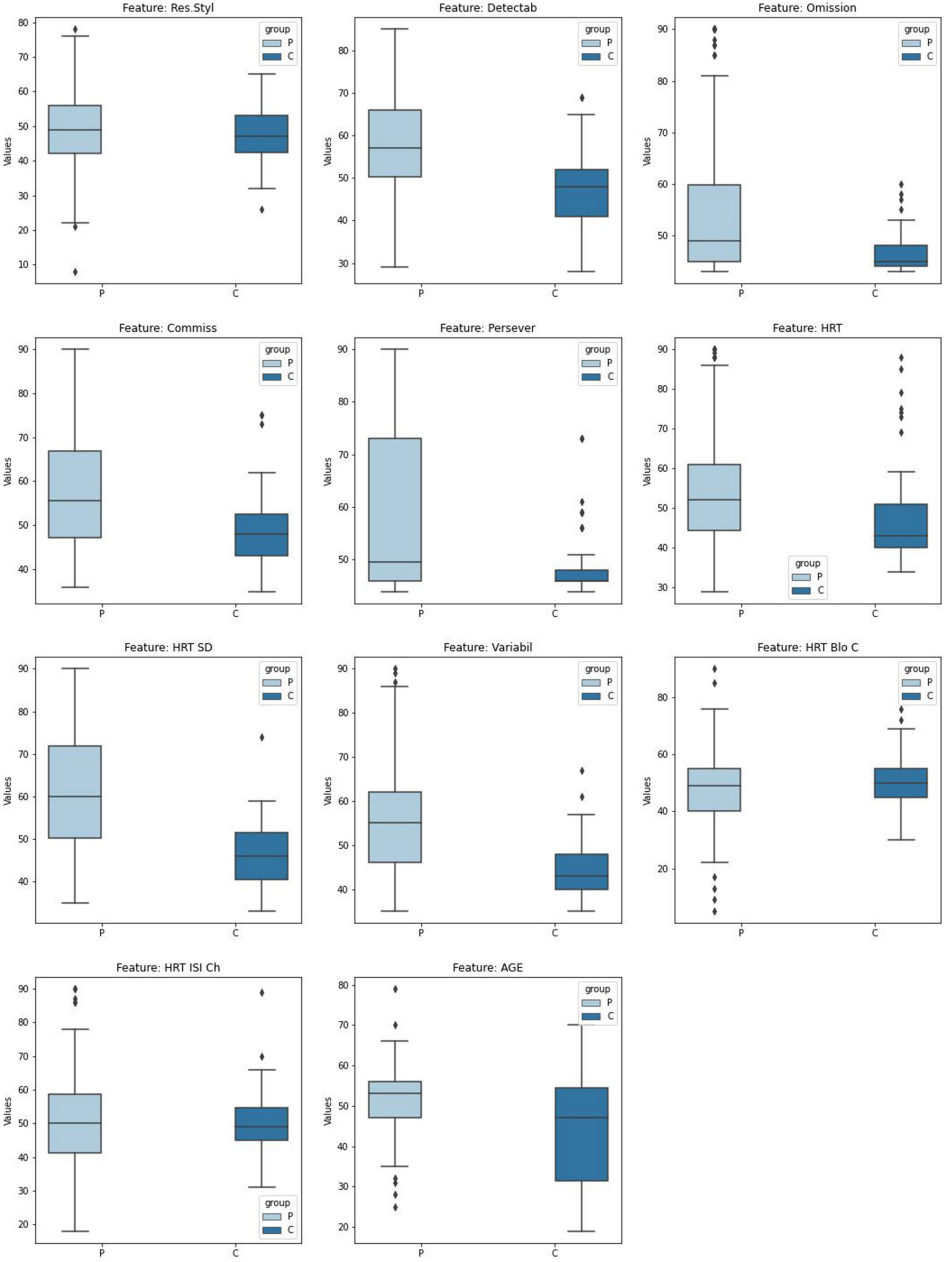
Box plot numerical features analysis by groups. Box plots for continuous variables by groups. The horizontal axis shows the groups, and the vertical axis shows the variable analyzed.

Box plots show for continuous variables the differences between quartiles between the different groups, including outliers. It is interesting to note the differences in some variables, although their significance was calculated using an independence test, such as omission (Omission N), perseverance (Persever N) or variability (Variabil N), are very evident in Figure 2.

Spearman’s rho value is calculated for the continuous variables to define the graph illustrating the relationship between them. Each edge of the graph will correspond to a value greater than 0.5 from Table 4. The *p*-value is added to check the significance of the rho value.

**Table 4:** Rho values > abs(0.5) for variables with high correlation in group P (diagnosed ME/CFS).

Features		Rho	*p*-Value
Detectab N	Commiss N	0.820382	0.00000000
HRT SD N	Variabil N	0.786083	0.00000000
Detectab N	Omission N	0.776174	0.00000000
Omission N	Variabil N	0.766189	0.00000000
Res.Styl N	HRT N	0.722694	0.00000000
Omission N	HRT SD N	0.691635	0.00000000
Detectab N	Variabil N	0.689658	0.00000000
Detectab N	Persever N	0.673743	0.00000000
Detectab N	HRT SD N	0.614872	0.00000000
Omission N	Persever N	0.609529	0.00000000
Persever N	Variabil N	0.590245	0.00000000
Persever N	HRT SD N	0.531106	0.00000000
Res.Styl N	Omission N	0.521658	0.00000000
HRT N	HRT SD N	0.501458	0.00000000
Res.Styl N	Commiss N	−0.563669	0.00000000

The undirected graph is constructed with the data from Table 4 using the rho value itself as the weight on each edge, and the result is shown in Figure 2.

As a result, three variables are found to be unrelated. In comparison, the five variables on the left of the graph show a complete regular graph as a pentagon, i.e., all five variables are related. The thicker the edge, the higher the correlation, and the thinner and redder the values close to 0.5.

### Features importance random forest based

**Figure 3:**
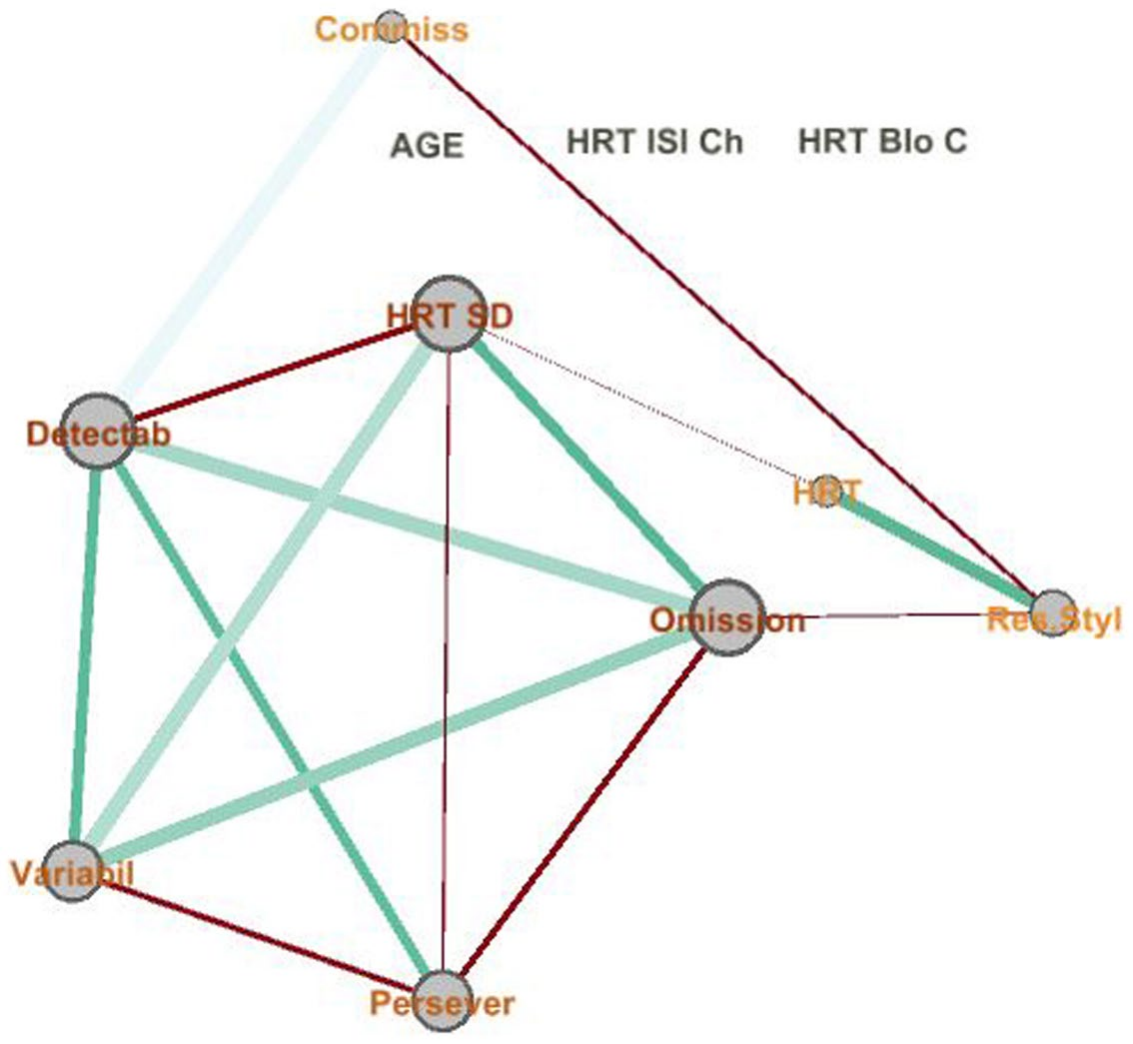
Network relationship between numerical features. The graph represents each continuous variable as a node, indicating the size of the node as a function of degree (the number of edges). Both the size and color depend on Spearman’s rho value.

The classification algorithm is run in a Python environment (v 3.7.14), and the library used is sklearn (v 1.1). Random forest is a Supervised Machine Learning Algorithm that is used widely in classification and regression problems. This model classifies if a patient is in a control group or not. The database is divided into 75% to train the model and provide a generalization to predict, based on the values of the continuous and categorical variables, whether it belongs to group P or group C. The remaining 25% is kept for testing the model, i.e., the model is run and compared to see how well the model fits the sample. The model offers 77.19% accuracy, which means the 25% test dataset predicted with 77.19% accuracy. After dependence analysis for continuous features, the most important were HRT N, Detecta N, and Variability. Both were found to be able to classify ME/CFS patients shown in Figure 3.

A threshold of 60 is identified in the variable Variability N. For the values above, it is rare to find patients in the control group, which could indicate a risk of cognitive impairment for patients diagnosed with ME/CFS. The results of the random forests showed that the CPT3™ could discriminate between presentations, mainly for the inattentive presentation.

**Figure 4:**
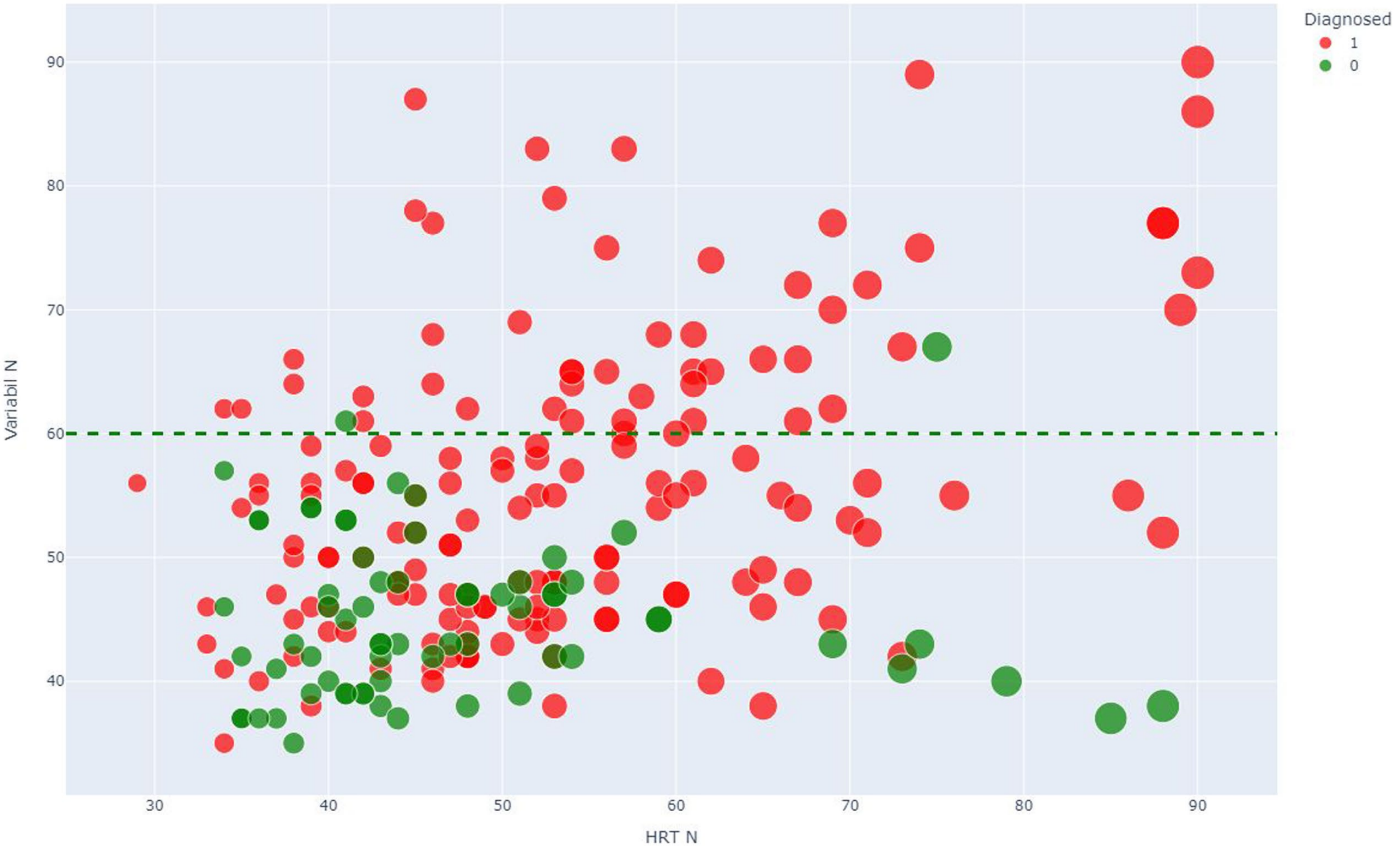
Scatter plot showing the discriminant threshold. Scatter plot of Variabil N (vertical axe) and HRT N (horizontal axe) where number 1 (red color) corresponds to P group, and number 0 (green color) corresponds to C group.

Figure 4 shows the importance of each variable. HRT N and VARIABILITY N were the critical variables. In contrast, other variables related to HRT, such as HRT by Block and HRT ISI Change, had less importanc**e** in discriminating ME/CFS presentation.

Moreover, the results of the CPT3™ showed that a relevant number of ME/CFS patients were associated with a likelihood of having a disorder characterized by attention deficits, such as ADHD.

## Discussion

Together with comprehensive diagnostic interviews, neuropsychological assessments could give the patient the “gold standard” for diagnosing ME/CFS. To the best of our knowledge, this is the first study assessing cognitive dysfunction in ME/CFS with CPT3™. It has been found that CPT3™ could detect cognitive impairments in all their main attentional measures (inattentiveness, impulsivity, sustained attention, and vigilance) in our ME/CFS individuals compared to healthy controls. More specifically, patients had a worse indicator of inattention, sustained attention, vigilance, impulsivity, and slow reaction time, showing the likelihood of having an ADHD-like pattern of functioning. In addition, relevant correlations were obtained between CPT3™ measures in this ME/CFS group. Accordingly, the adoption of the CPT3™ is supported as a specific test in ME/CFS aimed at analyzing the cognitive domains (attention and response speed) that seem to be key in the cognitive dysfunction of ME/CFS.

This work is in line with another previous study that assessed different types of attentional impairments in ME/CFS but using another brief and simple instrument (The Toulouse-Piéron Test) that allows the measurement of maintained attention—concentration, and resistance to monotony, and also, evaluates the multidimensional domain of attention, classified into different types such as arousal attention (alertness/activation), focused attention (detection of a stimulus) and sustained attention (attention to a stimulus or task for a prolonged time). These types of attention problems compromise various neuroanatomic structures, pathways, neurotransmitters, and their receptors. Their results support the reliability of maintained attention as a biomarker of ME/CFS, and attention deficit is a significant disability in patients affected by central fatigue. This neurocognitive dysfunction points to the neural networks involved in attention and focuses the pathological substrate in areas like the anterior cingulate cortex, lateral ventral prefrontal cortex, basal ganglia, or locus coeruleus (Murga et al., 2021).

Our results are in agreement with other neurocognitive research in ME/CFS reflecting attentional impairments on cognitive performance in these patients (Murga et al., 2021). Following these current findings, it would be quite interesting to monitor these brain areas with advanced techniques, such as fMRI, PET-scan, etc., to establish the key neurological bases that could be involved in CFS.

A neurocognitive profile in ME/CFS has not been described only because of the heterogeneity of the symptoms, but also for other shortcomings such as the lack of specific neuropsychological tests to evaluate it. CPT3™ represents a reliable alternative for assessing attention disorders in ME/CFS and allows a comprehensive measurement of multiple cognitive variables quickly. As a computerized test in the form of a computer game, the administration is more practical and stimulating for these patients, and the elaboration and quantification of scores are accurate. The Conners Continuous Performance Test (CPT) is widely used in clinical practice for its usefulness in the study of attention in various pathologies such as Attention Deficit Hyperactivity Disorder (ADHD; Newcorn et al., 2001; Baggio et al., 2020), although a previous study reported that the CPT3™ might be sensitive only to some of the core deficits of ADHD, but not hyperactivity. This instrument is not considered specific for ADHD, and although CPT may not differentiate between psychiatric and neurological disorders that result in executive dysfunctions (Baggio et al., 2020), it can be used in other diseases.

CPT3™ makes corrections for age and gender, so published results have already been corrected for biases identified in several studies (Newcorn et al., 2001; Conners et al., 2003; Seidman et al., 2005; Burton et al., 2010; Ramtekkar et al., 2010). T-scores are relative to age group and gender, with sex-independent consultation being possible as an option. Although the study design did not take into account a gender or age balance in the sample, and this should be taken into account in future studies, the gender variable was surprisingly significant in the Chi-square test. Furthermore, despite the fact that there is also a gender imbalance, especially in the group of diagnosed patients, the scores are in line with other studies that report on the influence of gender on chronic fatigue (Lim et al., 2020).

CFS/ME patients have more difficulty discriminating between targets and non-targets, and this poor detectability indicates inattention. A very unusual number of omission errors may indicate clinical impairment, fatigue, poor understanding of instructions, or a lack of motivation to respond with full effort. The results of the Mann–Whitney *U* test show that all variables are significant except HRT Block Change (HRT Blo C, meaning that the slope of HRT change in the six test blocks) and HRT ISI change [HRT ISI Ch N; indicates that the HRT change’s slope in the three ISIs (1, 2, and 4 s)]. A T-score of 60–69 in HRT is classified as slow response, and a T-score of 60–69 in Variability is interpreted as under-average performance. Slower reaction times and high inconsistency of response speed may be associated with the inattentive profile. High response speed variability indicates that the respondent’s attention and information processing efficiency varied throughout the administration (Kao and Thomas, 2010).

**Figure 5:**
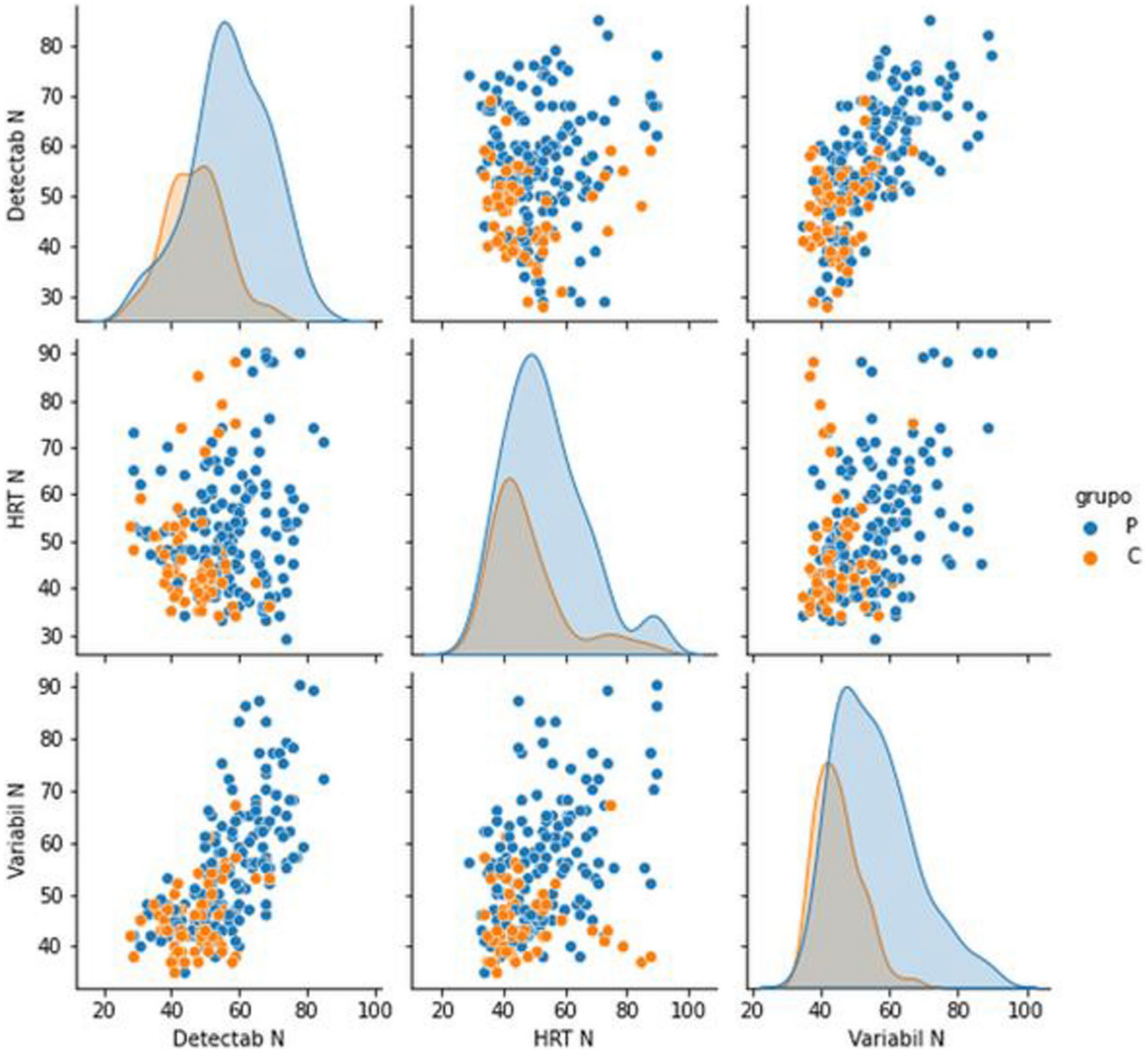
Pair plot analysis of the most significant features to discriminate between groups. Histograms as a function of the group and scatter plots of the combinations between the three chosen variables.

Indicators that measure response speed act as good discriminants between both groups. Hit Reaction Time (HRT) is the average response speed of correct responses for the entire administration, measured in milliseconds, for all non-perseverative responses made during the entire administration. An atypically slow HRT may indicate inattention, especially when error rates are high, but may also result from a very conservative response style. Variability, like HRT SD, is a measure of response speed consistency; however, Variability is a “within-respondent” measure (i.e., the amount of variability the respondent exhibited in 18 separate sub-blocks of the administration relative to their overall HRT SD score). Although Variability is a different measure than HRT SD, the two measures typically produce comparable results, and both are related to inattention. High response speed variability indicates that the respondent’s attention and processing efficiency varied throughout the administration. These scores mean that the response speed in the ME/CFS significantly differed from the response speed in the control group. The high variability of the response speed and the slow reaction time obtained indicate that ME/CFS suffer dysfunctions in the efficiency of information processing and commit more errors of omission, commission, and perseverance than healthy controls, presenting not only more attention problems, but being slower and with greater inconsistency in response speed when performing this test. The high variability of response speed indicates that the attention and processing efficiency of the ME/CFS patients varied throughout the administration. Figure 5 suggests that this is a discriminant element in differentiating ME/CFS from healthy people. The Hit Reaction Time Standard Deviation (HRT SD) measures the consistency of the speed of response to target stimuli across the administration. A high HRT SD in CFS/ME patients also indicates a greater inconsistency in response speed. It is sometimes indicative of inattention, which could suggest that ME/CFS patients were less engaged and processed stimuli less efficiently than healthy subjects during some parts of the CPT3^™^ administration. Overall, the ME/CFS group has more-atypical T-scores, which is associated with a high likelihood of having a disorder characterized by attention deficits, such as ADHD. However, assessors should keep in mind that other psychological and/or neurological conditions with attention-disrupting symptoms may also generate atypical scores (and thus a high or very high probability estimate). A previous study suggested that ADHD may be common in ME/CFS patients and is associated with a more severe psychopathology clinical profile (Sáez-Francàs et al., 2012).

For all the above, CPT3^™^ represents a reliable alternative to objectively and comprehensively assess attentional deficits in CFS. However, it also allows the assessment of other executive function domains, such as information processing speed and inhibition, providing the reaction time measures which can be considered discriminant indicators of ME/CFS patients. This tool makes it possible to assess the severity of cognitive dysfunction in this population and could improve the diagnosis and/or monitor these patients.

It should be noted that for a proper interpretation of CPT3™ test results in patients diagnosed with CFS, it would be necessary to investigate the clinical history further to assess the individualized performance of each measured dimension. The impact of medication use on cognitive functioning has not been explored in-depth, and it would be interesting to consider it in future studies. Even though the CPT lacks ecological validity (Baggio et al., 2020) as it does not adequately simulate the difficulties patients may experience in their daily life (i.e., it is free of external distractions that are likely to impair the patient’s real-life performance and it is a rather short task to represent overall performance), ME/CFS patients presented altered CPT3^™^ parameters when compared to healthy people. Most patients were not referred from primary care but belonged to a specialized unit in a tertiary care hospital. They probably have more mental and non-mental comorbidities, more severe symptoms, and more years of diagnosis. It should be considered to analyze the relationship between the results obtained through the CPT3^™^ with other variables, such as the severity of symptoms, associated comorbidities, and the time of diagnosis of ME/CFS, evaluating the changes over time in these participants. Using the test–retest after an estimated suitable time could be convenient for contemplating the evolution of cognitive functioning in these patients. Furthermore, it would be very interesting to analyze the relationship between the degree of fatigue, the psychopathological symptoms, and the cognitive performance of the CPT3™ in these patients.

Finally, it would be highly recommended in future studies to contemplate an analysis of how the presence of comorbid psychopathological symptoms in ME/CFS influences the cognitive performance obtained in the CPT3^™^, since it is a test that does not discriminate the presence of these factors. Furthermore, it would be interesting to replicate this study by including another group with severe psychiatric disorders like major depressive disorder.

### Limitations

All the patients come from the specialized chronic fatigue unit of the Vall D’Hebrón University Hospital in Barcelona. Patient data from other CFS-specialized units with similar protocols could enrich this work. Further research is needed to determine differences in ME/CFS patients to validate this work. CPT3™ response values have also not been evaluated based on controlled medication. There is no information on the influence of COVID-19 infection on the results in either group.

## Conclusion

CPT3^™^ adequately identifies ME/CFS in this clinical sample of adult participants compared to healthy controls. This profile of cognitive dysfunction could be related to other pathophysiological phenomena of CFS, and its determination could be key to elucidating the underlying basis and providing empirical evidence for the usefulness of this computerized neuropsychological test. Additionally, fatigue is the most common symptom associated with NCDs. There is insufficient evidence examining the relationship between fatigue and cognitive impairments in patients with other chronic diseases in which the symptom fatigue appears (e.g., major depressive disorder, fibromyalgia, postcovida, etc.). Further studies using CPT3^™^ could be useful to examine possible relationships between these two symptoms.

This study shows that a relevant number of ME/CFS patients had CPT3^™^ values compatible with a likelihood of having a disorder characterized by attention deficits, suggesting the possible existence of an underlying neurological basis for attentional dysfunction among the study patients. Furthermore, it was shown that two measures of response speed (hit reaction time and variability) act as good discriminants between both groups. Taken together, CPT3^™^ is a helpful neuropsychological instrument for discriminating cognitive impairments in attention and response speed in CFS. Therefore, the results obtained here will allow us to justify the use of the CPT3^™^ in other investigations that explore the cognitive functioning of these patients, and even this computerized test could be considered as a possible candidate marker for ME/CFS. As this is the only study reporting CPT3^™^ scores in adult ME/CFS patients, future studies are needed to compare this test across ME/CFS centers.

## Data availability statement

The raw data supporting the conclusions of this article will be made available by the authors, without undue reservation.

## Ethics statement

The studies involving human participants were reviewed and approved by Ethics Committee of the Vall d’Hebron University Hospital with the registration code PR (AG)257/2021. The patients/participants provided their written informed consent to participate in this study.

## Author contributions

All authors have participated in the conception and design of the study, and in the collection, analysis and interpretation of the data, as well as in the writing, revision and approval of the submitted manuscript.

## Conflict of interest

JR-Q was on the speakers’ bureau and/or acted as a consultant for Janssen-Cilag, Novartis, Shire, Takeda, Bial, Shionogi, Sincrolab, Novartis, BMS, Medicine, and Rubió Uriach, Technofarma, and Raffo in the last 3 years. He also received travel awards (air tickets + hotel) for taking part in psychiatric meetings from Janssen-Cilag, Rubió Shire, Takeda, Shionogi, Bial, and Medice. The Department of Psychiatry chaired by him received unrestricted educational and research support from the following companies in the last 3 years: Janssen-Cilag, Shire, Oryzon, Roche, Psious, and Rubió AR-U has received travel awards (air tickets + hotel) for taking part in annual psychiatric meetings from Lundbeck and has acted as a speaker at various training courses financed by Organon, Janssen-Cilag, and Lundbeck.

The remaining authors declare that the research was conducted in the absence of any commercial or financial relationships that could be construed as a potential conflict of interest.

## Publisher’s note

All claims expressed in this article are solely those of the authors and do not necessarily represent those of their affiliated organizations, or those of the publisher, the editors and the reviewers. Any product that may be evaluated in this article, or claim that may be made by its manufacturer, is not guaranteed or endorsed by the publisher.
